# Genome-wide miRNA profiling in plasma of pregnant women with down syndrome fetuses

**DOI:** 10.1007/s11033-020-05545-w

**Published:** 2020-05-30

**Authors:** Iveta Zedníková, Blanka Chylíková, Ondřej Šeda, Marie Korabečná, Eva Pazourková, Miroslav Břešťák, Miroslava Krkavcová, Pavel Calda, Aleš Hořínek

**Affiliations:** 1 Institute of Biology and Medical Genetics of the First Faculty of Medicine, Charles University and General University Hospital, Prague, Czech Republic; 2 Department of Obstetrics and Gynecology of the First Faculty of Medicine, Charles University and General University Hospital, Prague, Czech Republic; 3 Screening Center ProfiG2, Prague, Czech Republic; 4 GENvia Genetic Laboratories, Prague, Czech Republic; 5 3rd Department of Medicine, Department of Endocrinology and Metabolism, First Faculty of Medicine, Charles University and General University Hospital, Prague, Czech Republic

**Keywords:** Down syndrome, Fetal aneuploidy, Trisomy 21, Liquid biopsy, miRNA, NIPT

## Abstract

Down syndrome (DS) is one of the most common causes of intellectual disability and new approaches allowing its rapid and effective prenatal detection are being explored. In this study, we investigated the diagnostic potential of plasma microRNAs (miRNAs). This study builds upon our previous study in DS placentas, where seven miRNAs were found to be significantly up-regulated. A total of 70 first-trimester plasma samples from pregnant women were included in the present study (35 samples with DS fetuses; 35 with euploid fetuses). Genome-wide miRNA profiling was performed in the pilot study using Affymetrix GeneChip™ miRNA 4.1 Array Strips (18 samples). Selected miRNAs were then analysed in the validation study using quantitative reverse transcription PCR (RT-qPCR; 52 samples). Based on the current pilot study results (12 miRNAs), our previous research on chorionic villi samples (7 miRNAs) and the literature (4 miRNAs), a group of 23 miRNAs was selected for the validation study. Although the results of the pilot study were promising, the validation study using the more sensitive RT-qPCR technique and a larger group of samples revealed no significant differences in miRNA profiles between the compared groups. Our results suggest that testing of the first-trimester plasma miRNAs is probably not suitable for non-invasive prenatal testing (NIPT). Different results could be theoretically achieved at later gestational ages; however, such a result probably would have limited use in clinical practice.

**Electronic supplementary material:**

The online version of this article (10.1007/s11033-020-05545-w) contains supplementary material, which is available to authorized users.

## Background

Trisomy 21 (Down syndrome; DS) is the most common chromosomal disorder with an incidence of about 1:1000 to 1:1100 live births worldwide [1]. Virtually all DS patients suffer from cognitive impairment of various degree and craniofacial abnormalities. Other phenotypic characteristics, such as cardiovascular defects, childhood leukemia, gastrointestinal anomalies or early-onset Alzheimer’s disease, occur with various frequencies and exhibit interindividual heterogeneity [2]. It is generally accepted that the DS phenotype is caused by the excess genetic material of chromosome 21 (Hsa21); however, specific molecular mechanisms or pathways leading to particular DS features have not been found [3].

Many studies have focused on gene expression in various DS biological samples (for example, fetal or placental tissues, amniotic fluid cells, fetal or maternal blood) and they have reached varied conclusions. Some of these expression studies have even concluded that Hsa21 genes in DS are not significantly more expressed than other disomic genes [4–6].

The hypothesis that the major or most serious DS manifestations are caused by a few genes located at a relatively small region on Hsa21, the so-called Down Syndrome Critical Region (DSCR), was frequently discussed in previous years [7–9]. After reanalysis of all documented cases with partial trisomy 21 (PT21), the presumed DSCR was limited to a highly restricted DSCR (HR-DSCR), which does not contain any known gene [10].

Thus, all observations suggest that the DS phenotype is a consequence of global misregulation of gene expression, which occurs mainly due to imbalanced interactions between trisomic and disomic genes but also reflects the variability of the overall individual genome [11, 12]. Greater impact is exerted by haploinsufficient genes, which show a recognisable phenotype after the loss of one allele [13]. These genes, for example, dual-specificity tyrosine phosphorylation regulated kinase 1A (*DYRK1A)*, are also sensitive to three copies [14, 15]. Moreover, individual genomic and epigenomic backgrounds, including microRNA (miRNA) gene expression regulation, probably contribute to the final DS phenotype [16].

miRNAs are small (17–25 nucleotides) non-coding RNAs, which regulate gene expression at the post-transcriptional level. In most cases, the binding of miRNA to target mRNA with partial complementarity induces inhibition of translation (RNA silencing). Otherwise, when a high degree of complementarity between miRNA and its target is achieved, the mRNA is degraded [17]. More than 2600 human miRNAs have been described to date [18]. However, it has been found that one miRNA may affect hundreds of mRNAs; as a result, miRNAs regulate virtually all cellular processes. Therefore, miRNAs have been studied in the context of various pathologies, including cancer, cardiovascular diseases, diabetes and autoimmune diseases [19–22]. For example, miR-21 has been shown to play an essential role in various autoimmune diseases [23]. Furthermore, four miRNAs (miR-23a-3p, miR-27a-3p, miR-142-5p and miR-376c-3p) have been identified as sensitive non-invasive discriminators of early-stage colon cancer; two of these miRNAs (miR-23a-3p, miR-376c-3p) can also be used as prognostic markers [24].

Extracellular miRNAs associated with pregnancy are also systematically investigated in the plasma of pregnant women. Although differential miRNAs profiles have been described in many pregnancy-related conditions (for example, preeclampsia, preterm delivery, ectopic pregnancy, gestational diabetes mellitus and fetal trisomies) in comparison with normal pregnancies, the specific mechanisms of miRNA release into the maternal circulation are not fully elucidated [25, 26]. While most authors presume placental origin of these miRNAs as is the case for cell-free DNA, some also suggest a possible fetal origin [27, 28].

The present study follows on from our previous study comparing miRNA expression profiles in euploid and trisomic placentas. Seven miRNAs were found to be significantly up-regulated in DS placentas, three of which were located on chromosome 21 [29]. Because miRNAs are released via vesicles from the placenta to the maternal circulation, in the current study we focused on plasma samples of pregnant women bearing DS or euploid fetuses. We aimed to further investigate the biological functions of miRNAs and to explore their potential for non-invasive prenatal testing (NIPT) [30, 31].

## Results

### Pilot study

**Fig. 1:**
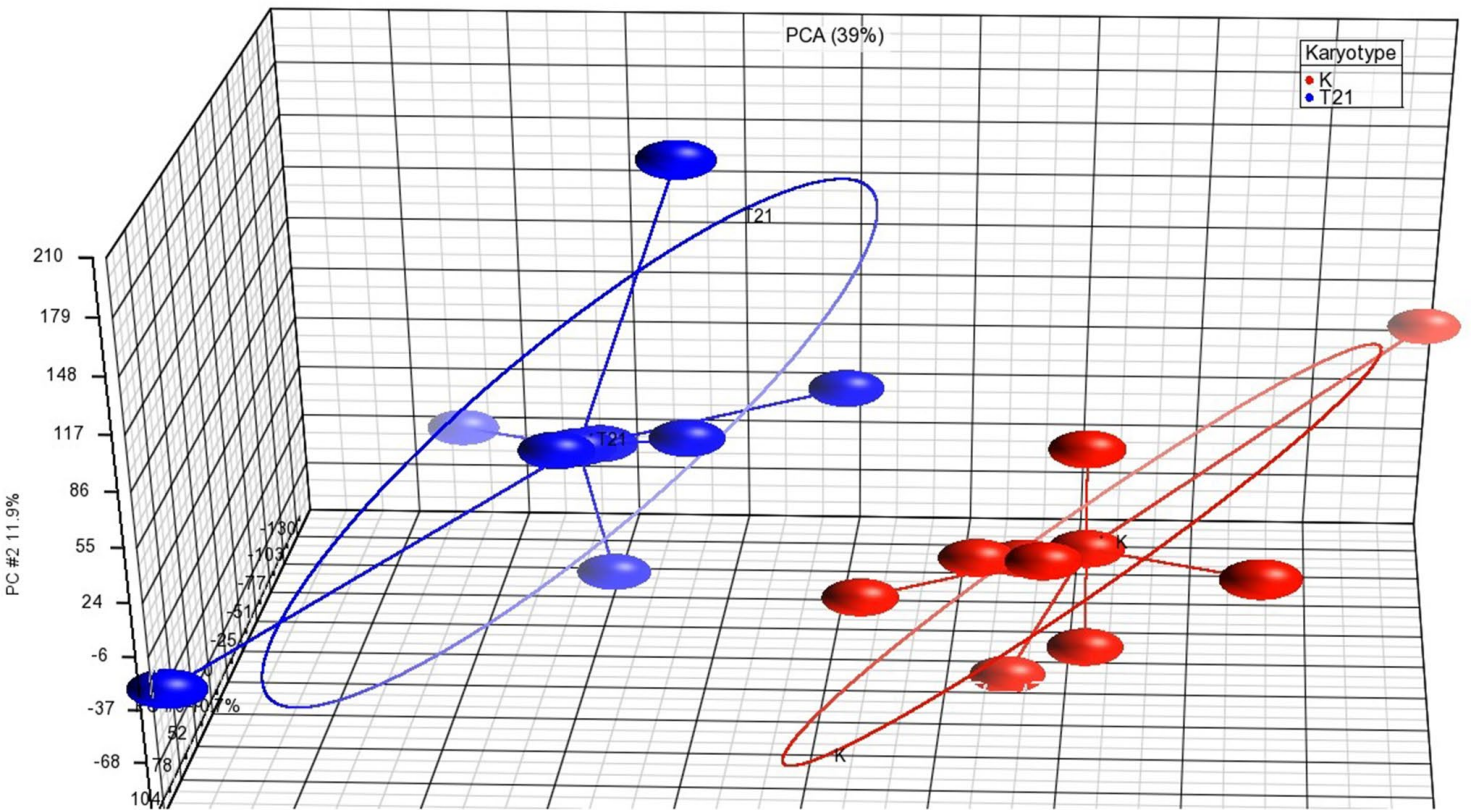
Scatter plot displaying compared groups of samples after principal component analysis (PCA). Samples with fetal trisomy (blue) are clearly separated from samples with euploid fetuses (red). (Color figure online)

The initial pilot study served to identify a wider panel of potentially dysregulated miRNAs in plasma of pregnant women with DS fetuses. Genome-wide analysis, which allows the determination of expression levels of all miRNAs listed in mirBase v.20 in one reaction, was selected. A total of 18 plasma samples (nine with fetal trisomy 21; nine with normal karyotype) were loaded into Affymetrix miRNA array strips. There was a clear separation of the compared groups of samples based on principal component analysis (PCA) (Fig. 1).

Twelve miRNAs which most significantly discriminated the two compared groups of samples were selected using one-way ANOVA (p-value ≤ 0.05; FC ≥ 1.5; Table 1). Half of these miRNAs were up-regulated in samples with fetal trisomy, half of them were down-regulated. None of the 12 selected miRNAs is on chromosome 21. Seven miRNAs that were validated as significantly elevated in DS placentas in our previous study [29] were not among the differentially expressed miRNAs in the current pilot study using plasma samples.

**Table 1:** Set of 23 miRNAs selected for the validation study

Selection based on		MirBase accession no	Systematic ID	Up/down-regulated
Validated results of previous study on CVS		MIMAT0000097	miR-99a	Up-regulated
		MIMAT0003340	miR-542-5p	Up-regulated
		MIMAT0000254	miR-10b	Up-regulated
		MIMAT0000423	miR-125b	Up-regulated
		MIMAT0003783	miR-615	Up-regulated
		MIMAT0000064	hsa-let-7c	Up-regulated
		MIMAT0003330	miR-654	Up-regulated
Current pilot study on plasma samples (Affymetrix miRNA 4.1 array strips); p ≤ 0.05	p ≤ 0.05 + FC ≥ 1.5	MIMAT0017991	hsa-miR-3613-3p	Up-regulated
		MIMAT0000062	hsa-let-7a-5p	Down-regulated
		MIMAT0000065	hsa-let-7d-5p	Up-regulated
		MIMAT0019745	hsa-miR-4668-5p	Down-regulated
		MIMAT0000421	hsa-miR-122-5p	Up-regulated
		MIMAT0002871	hsa-miR-500a-3p	Up-regulated
		MIMAT0000732	hsa-miR-378a-3p	Up-regulated
		MIMAT0005929	hsa-miR-1275	Down-regulated
		MIMAT0004614	hsa-miR-193a-5p	Down-regulated
		MIMAT0025478	hsa-miR-6511a-5p	Down-regulated
		MIMAT0027682	hsa-miR-6891-5p	Down-regulated
		MIMAT0004983	hsa-miR-940	Up-regulated
	+ literature	MIMAT0005898	hsa-miR-1246	–
		MIMAT0002824	hsa-miR-498	–
		MIMAT0000101	hsa-miR-103a-3p	–
		MIMAT0000104	hsa-miR-107	–

### Validation study

A total of 23 miRNAs were selected for the validation study (Table 1). Apart from the 12 miRNAs identified in the pilot study, the selection was based on the results of our previous study on placenta samples (seven miRNAs) and the results of the current pilot study in combination with information in the literature (four miRNAs) [28, 32–35].

The expression levels of the 23 selected miRNAs were determined using qPCR analysis with individual miRNA assays in an independent set of samples. A total of 52 plasma samples (26 with fetal trisomy 21; 26 with normal karyotype) were included. Differences in miRNA levels between compared groups of samples were evaluated using the non-parametric Mann–Whitney *U*-test (p-value ≤ 0.05; FC ≥ 2; Benjamini–Hochberg correction). None of the 23 tested miRNAs was confirmed to be significantly differentially expressed between plasma samples with fetal trisomy and samples with a confirmed normal fetal karyotype.

The power of the validation study, tested using post-hoc analysis in G*Power, [36] was high enough for all miRNAs tested (power > 0.98; table S2) with one exception – miR-542 (power = 0.103). This miRNA had been added to the validation set of miRNAs because it was dysregulated in trisomic placentas in our previous study, so we wanted to verify its plasma levels. miR-542 levels in the plasma of pregnant women are very low—either it was detected with a high Ct value or it was completely absent and therefore probably not applicable for diagnosis at the plasma level.

## Discussion

In our previous study, we performed miRNA expression profiling of chorionic villi samples (CVS) from euploid and trisomic pregnancies [29]. A total of 80 CVS samples (40 with normal karyotype, 40 with trisomy of chromosome 21) were included. Seven miRNAs were verified using qPCR as significantly up-regulated in DS placentas (miR-99a, miR-542-5p, miR-10b, miR-125b, miR-615, let-7c and miR-654). Of these, three miRNAs were located on chromosome 21 (miR-99a, miR-125b, let-7c). As well as genes involved in various essential biological processes, we identified many genes involved in placenta development (*GJA1*, *CDH11*, *EGF*, *ERVW-1*, *ERVFRD-1*, *LEP* and *INHA*) as being potentially altered by elevated miRNA levels.

Human placenta expresses more than 500 different miRNAs, some of them specific for this tissue [37]. Placental-specific miRNAs are expressed from three main clusters—C14MC (chromosome 14 miRNA cluster), C19MC and miR-371-3 [38]. Typical changes in the expression of miRNAs from these three clusters during pregnancy suggest their potential involvement in physiological processes [39]. For example, expression of miRNAs from cluster C19MC increases continually from the first to the third trimester and closely correlates with placenta growth [40]. Decreased levels of specific miRNAs from C19MC (hsa-miR-518b, hsa-miR-1323, hsa-miR-520 h, and hsa-miR-519d) have been associated with fetal growth restriction [41]. miRNAs are released from placenta, primarily from placental trophoblast, into maternal and fetal circulation mainly via exosomes [30]. Increased concentrations of both total exosomes and placenta-derived exosomes were found in the plasma of pregnant women who subsequently developed preeclampsia [42]. Moreover, two of these exosomal miRNAs (hsa-miR-486-1-5p and hsa-miR-486-2-5p) were suggested as potential markers for presymptomatic diagnosis of preeclampsia.

Our previous results suggested that miRNAs upregulated in DS placentas (miR-99a, miR-542-5p, miR-10b, miR-125b, miR-615, let-7c and miR-654) can potentially affect the expression of many genes crucial for intercellular communication (e.g. connexin 1), cytotrophoblast cell adhesion (cadherin 11) or syncytiotrophoblast differentiation (syncytin-1 and 2) and, thus, affect physiological placental development [29]. However, the placenta is not the only determinant of pregnancy-associated miRNA levels in maternal and fetal blood. Another source or mechanism influencing these levels is probably involved [31]. The hypothesis that miRNAs are somehow transported from the fetus into the maternal circulation and vice versa is still unproven [28].

To further extend our knowledge of the biological functions of miRNAs and assess their diagnostic potential, we focused the follow-up study on maternal plasma samples. To the best of our knowledge, this is the first study performing genome-wide miRNA profiling in plasma samples of pregnant women with euploid and DS fetuses.

Methods analysing genome-wide miRNA profiling (NGS or arrays) require a high miRNAs input, which is challenging in the case of plasma samples. Therefore, most of the studies analyse only a selected group of miRNAs in plasma using qPCR, where a much smaller input is needed, or perform a genome-wide analysis of whole maternal blood, where the overwhelming background from maternal blood cells makes it virtually impossible to analyse cell-free nucleic acids from the placenta [34].

To achieve the highest yield and purity of miRNAs from plasma for Affymetrix miRNA array strips, we performed exhaustive and systematic method optimisation (Materials and Methods; S1). Use of miRNA arrays enabled us to evaluate all miRNAs listed in miRBase v.20 in one reaction. Twelve miRNAs were identified as being significantly dysregulated between compared groups of samples.

Nevertheless, promising results from the initial study phase were not verified in subsequent validation phase using more sensitive method RT-qPCR and a larger group of samples.

**Fig. 2:**
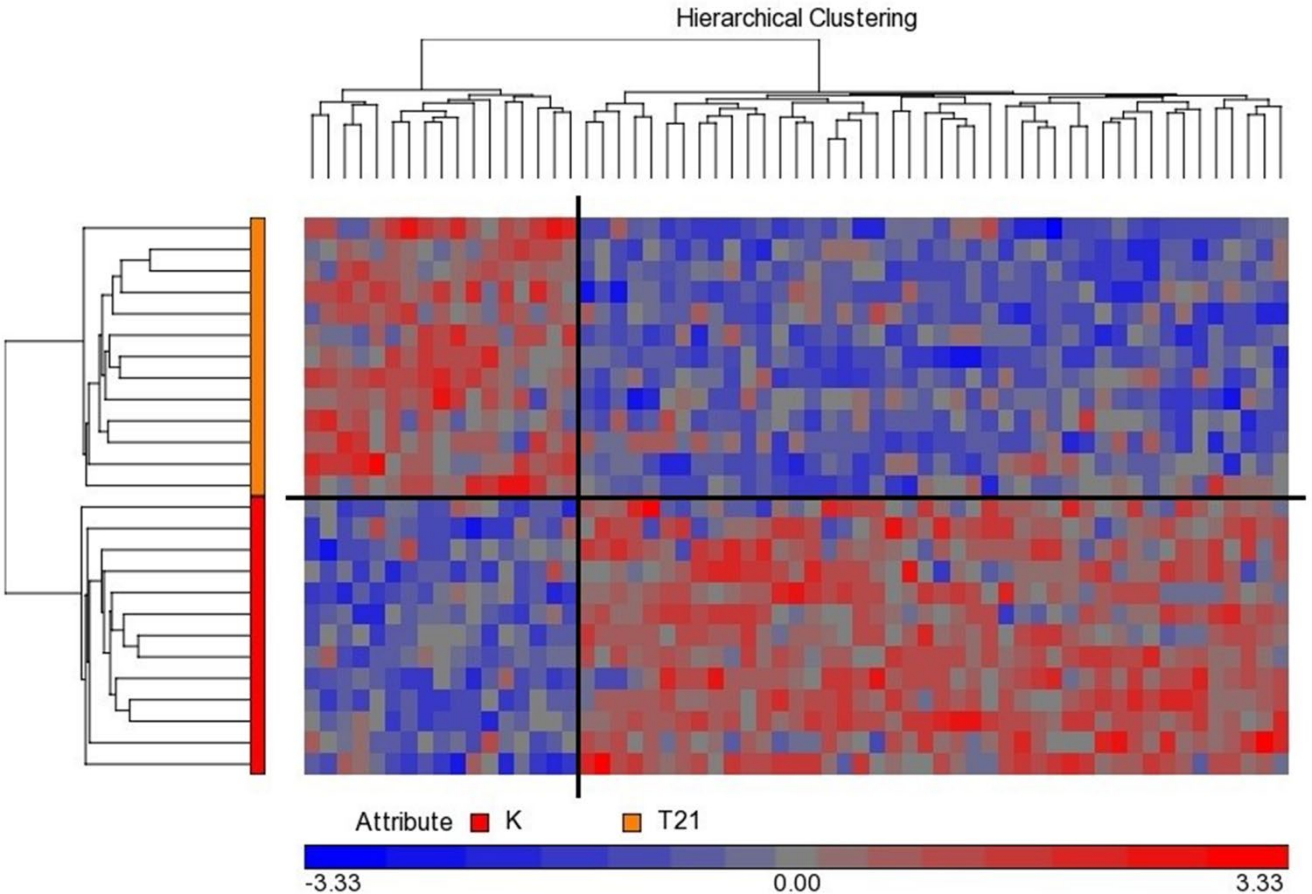
Heatmap displaying clear separation of pregnancies with DS fetuses (T21; orange) in comparison with controls (K; red) according to expressions of 61 miRNAs with the lowest p-value (fold change value was not considered). These data are based on the results of the pilot study (miRNA arrays). Most of the dysregulated miRNAs are down-regulated in the DS group of plasma samples (44 miRNAs). (Color figure online)

We could not select a single miRNA that would discriminate euploid and DS pregnancies on the plasma level. However, clear separation of compared groups is visible when comparing the levels of the larger group of most dysregulated miRNAs obtained from miRNA arrays (Fig. 2).

Several articles comparing miRNA levels in the plasma of pregnant women bearing euploid and DS fetuses have been published so far (Table 2). Nevertheless, these studies may have possibly come to different results due to the different workflow used. The lack of a standardised normalisation strategy represents a general issue in plasma miRNA evaluation. Various reference miRNAs are used for normalisation of raw expression data. For example, miR-16 is often selected as a reference target, but it was found to be very susceptible to hemolysis [43]. Small nuclear or nucleolar RNAs are suitable only for normalisation of samples where nuclear material is expected, but not for plasma samples [44]. On the other hand, global mean normalisation is applicable only for a larger miRNA set (> 100 miRNAs) [45]. To prevent distortion of our results, we decided to normalize our data with the same total miRNAs input as described previously [46].

**Table 2:** Overview of the articles on the topic

Study	Included miRNAs	Samples (C/T21)	GW	Normalization	Correction
Kotlabova et al.	5	12/12	18.5	miR-16; let-7d	Bonferroni
Erturk et al.	14	33/23	17–18	U6 snRNA	No
Zbucka-Kretowska et al.	800	12/12	15–18	Global mean	No
Kamhieh-Milz et al.	1043	7/7	14.1	Multiple strategies	No
Our study	All in miRBase v.20	35/35	13.3	Input volume	B-H

*C* controls, *T21* samples with fetal trisomy of 21, *GW* average gestational week, *B-H* Benjamini-Hochberg

Kotlabova et al. performed expression analysis of five miRNAs from chromosome 21 (miR-99a, let-7c, miR-125b-2, miR-155 and miR-802) using qPCR with normalisation to reference miRNAs—miR-16 and let-7d [47]. Nevertheless, they found no differences between selected miRNA levels in the compared groups of samples (12 pregnancies with DS fetuses; 12 control samples). Another study evaluating 14 miRNAs from Hsa21 (including the five miRNAs which Kotlabova et al. focused on) also using qPCR with normalisation to U6 snRNA was published by Erturk et al. [26]. They compared 33 euploid and 23 trisomic pregnancies and found two miRNAs—miR-99a and miR-3156—which were elevated in DS pregnancies. The most comprehensive study so far has been carried out by Kamhieh-Milz et al. [28]. A group of 1043 miRNAs were analysed using the high-throughput qPCR SmartChip Human miRNA Panel. Nevertheless, a very small number of samples were included (7 DS fetuses; 7 controls). Using a combination of three different normalisation strategies (corrected threshold cycle values, normalised relative quantities and a combination of both methods together) they found 36 miRNAs to be differentially expressed in DS versus control pregnancies, neither miR-99a nor miR-3156 were among them. The latest work on the topic was published by Zbucka-Kretowska et al. [48]. They examined levels of 800 miRNAs using NanoString technology within 12 DS pregnancies and 12 controls. Using normalisation to the geometric mean of top 100 probes (global mean), a group of 13 miRNAs was found to be dysregulated.

Although the study by Kotlabova et al. applied a correction for multiple testing, the remaining three studies did not. Omitting this correction can lead to false-positive results, especially for a high number of comparisons and small sample size, as in the case of the studies by Kamhieh-Milz et al. and Zbucka-Kretowska et al. [49]. Moreover, Zbucka-Kretowska et al. themselves reported that using Benjamini-Hochberg's correction they would not reach any statistically significant results.

Our study included samples from early gestational weeks (11–14), which would allow potential utilization of miRNA markers found for early NIPT. However, our results from the validation study demonstrate that levels of pregnancy-associated miRNAs are too low in such early pregnancies. Analysis of samples from later gestational weeks would potentially lead to different results, but without the required potential for early diagnosis. Differences in gestational age could also contribute to the discrepancies between the results of studies compared (Table 2).

As well as different detection platforms, various preanalytical steps in sample handling, like sample storage conditions (time, temperature), type of preservative tube, concrete blood centrifugation conditions for plasma separation, plasma input volume to isolation and type of miRNA isolation (see Supplement Materials; S1), have also proven to have an impact on the results achieved [43, 50, 51].

Regardless of the different procedures and data processing, none of the studies comparing plasma samples from euploid and DS pregnancies found any miRNA that could discriminate compared groups in all cases. These results indicate that miRNA determination in plasma from pregnant women is not applicable for NIPT of fetal DS.

Since most of the placental miRNAs are released to the circulation of pregnant woman via exosomes [30], it would be interesting in a future study to focus specifically on exosomal miRNAs. Exosomal miRNAs may be masked in the pool of total plasmatic miRNAs by other abundant miRNAs associated with RNA-binding proteins or derived from apoptotic cells. Exosomes are a specific subtype of extracellular vesicles, which probably play a significant role in the intercellular communication pathways involved in placentation, the formation of the vascular system between the mother and fetus and the induction of maternal immune tolerance to the fetus [52–55]. Therefore, exosomes could have potential as early non-invasive biomarkers of various pregnancy complications, especially those connected to placenta development. Indeed, exosomes are currently intensively studied in preeclampsia [42, 56]. As Down syndrome pregnancies are also complicated by abnormal placentation [57], exosomes released from such an impaired placenta could be also promising markers for early detection of Down syndrome fetuses from the maternal circulation. So far, miRNAs from circulating nanoparticles have only been studied in young individuals with DS and their siblings with promising results achieved [58].

## Conclusion

Previously, we found dysregulated miRNA levels in DS placentas that potentially interfere with essential biological pathways. In our current study, we focused on the plasma of pregnant women to explore whether overexpressed placental miRNAs are also detectable in maternal circulation and therefore applicable for NIPT. To our best knowledge, this is the first study performing genome-wide profiling of plasmatic miRNAs on such a large cohort of first-trimester pregnant women with DS fetuses. However, we could not conclusively demonstrate differences in miRNA levels in the first-trimester plasma of pregnant women with euploid and DS fetuses. The main reason probably was the high background of maternal miRNAs, which did not allow detection of potential differences in pregnancy-associated miRNAs in such early pregnancies. Further research will be needed to clarify the role of miRNAs in DS pathophysiology.

## Materials and methods

The study consisted of two phases. A pilot study was performed using Affymetrix GeneChip™ miRNA 4.1 Array Strips (Affymetrix, USA) and enabled the selection of a broader spectrum of miRNAs with different expression levels between the compared groups (18 samples of plasma of pregnant women included; nine with trisomic and nine with euploid fetuses). Based on the pilot study results, our previous research on CVS samples [29] and the literature, a group of 23 miRNAs were selected for the subsequent validation (Table 1). TaqMan Advanced miRNA Assays (Life Technologies, USA) were used in validation study (52 samples included; 26 with trisomic and 26 with euploid fetuses).

### Clinical samples

Plasma samples of pregnant women were collected between January 2015 and November 2017 at the Department of Obstetrics and Gynecology of the First Faculty of Medicine and General University Hospital in Prague, Screening Centre ProfiG2 in Prague and Genvia Genetic Laboratories. All samples were obtained before CVS between the 11th and 14th gestational weeks from patients with an increased fetal trisomy risk based on first trimester combined test, increased maternal age or abnormal ultrasound finding (only in case of DS pregnancies). A total of 70 samples were included in the study; 35 of them were cytogenetically confirmed to have complete fetal trisomy of chromosome 21 (47, XX, + 21 or 47, XY, + 21), and 35 to have normal karyotype (46, XX or 46, XY). Only non-smoking pregnant women without any medication or any subsequently identified placental pathologies (e.g., preeclampsia), which can affect the overall miRNA profile, were included in our study [59, 60]. There were no statistically significant differences between compared groups of samples in maternal age, BMI, fetal sex or sample storage time (Table 3). All of the participants included were Caucasians.

**Table 3:** Clinical characteristics of individuals included to the study

	Maternal age	BMI	Fetal sex		Gest. age	N
	Average ± SD	Average ± SD	Female	Male	Average ± SD	
Pilot study						
Euploid fetuses	34.8 ± 1.6	23.8 ± 3.1	4	5	13.3 ± 0.3	9
DS fetuses	36.9 ± 3.3	23.2 ± 2.1	4	5	13.2 ± 0.4	9
Validation study						
Euploid fetuses	33.3 ± 4.8	23.2 ± 3.8	13	13	13.4 ± 1.3	26
DS fetuses	34.4 ± 6.3	24.1 ± 4.5	10	12	13.5 ± 0.8	26

The study was approved by the Ethical Committee of the First Faculty of Medicine, Charles University and General University Hospital in Prague. The informed consent was obtained from all participants.

### Sample processing and miRNA isolation

#### Plasma separation and storage

Peripheral blood samples were collected by venepuncture using cell-free DNA BCT tubes (Streck, USA) to prevent coagulation. Tubes were stored at room temperature and processed within 6 h after sampling at the Institute of Biology and Medical Genetics of the First Faculty of Medicine and General University Hospital in Prague and Genvia Genetic Laboratories. Two-step centrifugation was performed to obtain plasma samples from peripheral blood samples: (1) 1100×*g* for 10 min at 10 °C and (2) 14,500×*g* for 10 min at room temperature. Plasma samples were frozen at − 80 °C.

#### Optimisation of miRNA extraction

To achieve the highest miRNA yield, we tested six different miRNA isolation kits with various input (200–2000 μl) and elution (14–50 μl) volumes. Improvement in miRNA extraction using a vacuum concentrator or glycogen were also tested. Concentration and quality of isolated miRNA samples were then evaluated using three different approaches (fluorometer, spectrophotometer and RT-qPCR). The best version of sample processing was selected for the preparation of clinical samples. The detailed optimisation procedure is provided in Supplemental information (S1).

#### miRNA isolation

Total RNA enriched for small RNAs was extracted from 900 μl of plasma using a NucleoSpin miRNA Plasma kit (Macherey–Nagel, Germany) and eluted with 20 μl of supplied elution buffer. The whole procedure was performed to achieve the highest yield based on previous optimisation (S1) and following manufacturer’s recommendations. Before proceeding to the microarray (pilot study) or reverse transcription (validation study) step, the miRNA concentration of all samples was measured using a fluorometer Qubit 3.0 (Thermo Fisher Scientific, USA) and total RNA concentration was determined by spectrophotometer (IMPLEN, Germany). While miRNA concentration of all samples ranged between 2 and 3 ng/μl, concentrations of total RNA were about ten times higher (20–30 ng/μl).

### miRNA expression analysis

#### Pilot study: genome-wide miRNA profiling

Total RNA (130 ng) enriched for low molecular weight RNA from each sample was labelled using the FlashTag Biotin HSR RNA Labelling Kit (Affymetrix) on the GeneAtlas Hybridization Station (Affymetrix) and subsequently, it was processed using the GeneAtlas Hybridization, Wash and Stain Kit for miRNA Array Strips (Applied Biosystems, USA) on the GeneAtlas Fluidics Station (Affymetrix) according to the manufacturer’s instructions. Array strip fluorescence intensities were finally determined using the GeneAtlas Imaging Station (Affymetrix). Raw data were processed and visualized using Partek Genomics Suite software (Partek, USA).

#### Validation study: qPCR using TaqMan miRNA assays

A total of 23 miRNAs were selected for the validation study (Table 1). The set of miRNAs consisted of three groups:

1. Seven miRNAs verified as being overexpressed in DS placentas in our previous study [29],
2. 12 miRNAs with significantly different expression levels between plasma samples of pregnant women with euploid and trisomic fetuses according to the results of the pilot study (p-value ≤ 0.05; fold change (FC) ≥ 1.5),
3. Four miRNAs which did not fulfil the above conditions but were reported in the literature as being possibly associated with DS pathophysiology [28, 32, 33].

The same total miRNAs input (4 ng) from each sample based on fluorometer (Qubit 3.0) measurement was reverse-transcribed using TaqMan™ Advanced miRNA cDNA Synthesis Kit (Applied Biosystems). Expression of each miRNA was determined using quantitative real-time PCR (qPCR) with TaqMan Advanced miRNA Assays and TaqMan Fast Advanced Master Mix (Applied Biosystems). We have followed the procedure recommended by the manufacturer. All reactions were performed in triplicate. Expression levels of the miRNAs were detected on QuantStudio 12 K Flex Real-time PCR System (Applied Biosystems).

### Data analysis

#### Pilot study

Raw results of miRNA array strips were evaluated using Partek Genomics Suite software (Partek, USA). One-way ANOVA with a cut-off p-value ≤ 0.05 and FC ≥ 1.5 was used for detection of differentially expressed miRNAs. To control the false discovery rate, the Benjamini–Hochberg correction was applied. All visualisations, such as heatmaps, were prepared using Partek software. Samples were normalised to the same total miRNA input based on measurement by fluorometer (Qubit 3.0).

#### Validation study

For the initial data processing, QuantStudio 12 K Flex Software v1.1.2 and ExpressionSuite software v1.0.3 (Thermo Fisher Scientific) were used. The qPCR results normalised to the same total miRNAs input as in the pilot study were statistically evaluated using qBase + v2.4 software (Biogazelle, Belgium). Expression data of plasma from women with euploid and trisomic fetus were compared using a nonparametric Mann–Whitney test with corrected cut-off p-value ≤ 0.05 and FC ≥ 2. To evaluate the power of our study we performed post-hoc analysis using G*Power software [36].

## Electronic supplementary material

Below is the link to the electronic supplementary material.

Supplementary file1 (DOC 33 kb)

Supplementary file2 (DOC 16 kb)

## References

[1] Organization WH. https://www.who.int/genomics/public/geneticdiseases/en/index1.html. Accessed 25 Jan 2018

[2] Gardiner K, Herault Y, Lott IT, Antonarakis SE, Reeves RH, Dierssen M (2010). Down syndrome: from understanding the neurobiology to therapy. J Neurosci.

[3] Patterson D (2009). Molecular genetic analysis of down syndrome. Hum Genet.

[4] Bianchi DW (2011). Gene expression analysis of amniotic fluid: new biomarkers and novel antenatal treatments. Clin Biochem.

[5] Slonim DK, Koide K, Johnson KL, Tantravahi U, Cowan JM, Jarrah Z, Bianchi DW (2009). Functional genomic analysis of amniotic fluid cell-free mRNA suggests that oxidative stress is significant in down syndrome fetuses. Proc Natl Acad Sci USA.

[6] Olmos-Serrano JL, Kang HJ, Tyler WA, Silbereis JC, Cheng F, Zhu Y, Pletikos M, Jankovic-Rapan L, Cramer NP, Galdzicki Z (2016). Down syndrome developmental brain transcriptome reveals defective oligodendrocyte differentiation and myelination. Neuron.

[7] Rahmani Z, Blouin J-L, Creau-Goldberg N, Watkins PC, Mattei J-F, Poissonnier M, Prieur M, Chettouh Z, Nicole A, Aurias A (1989). Critical role of the D21S55 region on chromosome 21 in the pathogenesis of down syndrome. Proc Natl Acad Sci.

[8] Olson L, Richtsmeier J, Leszl J, Reeves R (2004). A chromosome 21 critical region does not cause specific down syndrome phenotypes. Science.

[9] Jiang X, Liu C, Yu T, Zhang L, Meng K, Xing Z, Belichenko PV, Kleschevnikov AM, Pao A, Peresie J, Wie S, Mobley WC, Yu YE (2015). Genetic dissection of the down syndrome critical region. Hum Mol Genet.

[10] Pelleri MC, Cicchini E, Locatelli C, Vitale L, Caracausi M, Piovesan A, Rocca A, Poletti G, Seri M, Strippoli P (2016). Systematic reanalysis of partial trisomy 21 cases with or without down syndrome suggests a small region on 21q22.13 as critical to the phenotype. Hum Mol Genet.

[11] Ait Yahya-Graison E, Aubert J, Dauphinot L, Rivals I, Prieur M, Golfier G, Rossier J, Personnaz L, Creau N, Blehaut H, Robin S, Delabar JM, Potier MC (2007). Classification of human chromosome 21 gene-expression variations in down syndrome: impact on disease phenotypes. Am J Hum Genet.

[12] Kahlem P (2006). Gene-dosage effect on chromosome 21 transcriptome in trisomy 21: implication in down syndrome cognitive disorders. Behav Genet.

[13] Huang N, Lee I, Marcotte EM, Hurles ME (2010). Characterising and predicting haploin sufficiency in the human genome. PLoS Genet.

[14] Ji J, Lee H, Argiropoulos B, Dorrani N, Mann J, Martinez-Agosto JA, Gomez-Ospina N, Gallant N, Bernstein JA, Hudgins L (2015). DYRK1A haploinsufficiency causes a new recognizable syndrome with microcephaly, intellectual disability, speech impairment, and distinct facies. Eur J Hum Genet.

[15] Conrad B, Antonarakis SE (2007). Gene duplication: a drive for phenotypic diversity and cause of human disease. Annu Rev Genomics Hum Genet.

[16] Elton TS, Sansom SE, Martin MM (2014). Trisomy-21 gene dosage over-expression of miRNAs results in the haploinsufficiency of specific target proteins. RNA Biol.

[17] Meister G, Landthaler M, Dorsett Y, Tuschl T (2004). Sequence-specific inhibition of microRNA-and siRNA-induced RNA silencing. RNA.

[18] Kozomara A, Birgaoanu M, Griffiths-Jones S (2018). miRBase: from microRNA sequences to function. Nucleic Acids Res.

[19] Yuan T, Huang X, Woodcock M, Du M, Dittmar R, Wang Y, Tsai S, Kohli M, Boardman L, Patel T, Wang L (2016). Plasma extracellular RNA profiles in healthy and cancer patients. Sci Rep.

[20] Zhou SS, Jin JP, Wang JQ, Zhang ZG, Freedman JH, Zheng Y, Cai L (2018). miRNAS in cardiovascular diseases: potential biomarkers, therapeutic targets and challenges. Acta Pharmacol Sin.

[21] Karolina DS, Armugam A, Sepramaniam S, Jeyaseelan K (2014). miRNAs and diabetes mellitus. Expert Rev Endocrinol Metab.

[22] Pauley KM, Cha S, Chan EK (2009). MicroRNA in autoimmunity and autoimmune diseases. J Autoimmunol.

[23] Wang S, Wan X, Ruan Q (2016). The MicroRNA-21 in autoimmune diseases. Int J Mol Sci.

[24] Vychytilova-Faltejskova P, Radova L, Sachlova M, Kosarova Z, Slaba K, Fabian P, Grolich T, Prochazka V, Kala Z, Svoboda M, Kiss I, Vyzula R, Slaby O (2016). Serum-based microRNA signatures in early diagnosis and prognosis prediction of colon cancer. Carcinogenesis.

[25] Zhao Z, Moley KH, Gronowski AM (2013). Diagnostic potential for miRNAs as biomarkers for pregnancy-specific diseases. Clin Biochem.

[26] Erturk B, Karaca E, Aykut A, Durmaz B, Guler A, Buke B, Yeniel AO, Ergenoglu AM, Ozkinay F, Ozeren M, Kazandi M, Akercan F, Sagol S, Gunduz C, Cogulu O (2016). Prenatal evaluation of MicroRNA expressions in pregnancies with down syndrome. Biomed Res Int.

[27] Miura K, Miura S, Yamasaki K, Higashijima A, Kinoshita A, Yoshiura K-i, Masuzaki H (2010). Identification of pregnancy-associated MicroRNAs in maternal plasma. Clin Chem.

[28] Kamhieh-Milz J, Moftah RF, Bal G, Futschik M, Sterzer V, Khorramshahi O, Burow M, Thiel G, Stuke-Sontheimer A, Chaoui R, Kamhieh-Milz S, Salama A (2014). Differentially expressed microRNAs in maternal plasma for the noninvasive prenatal diagnosis of down syndrome (trisomy 21). Biomed Res Int.

[29] Svobodova I, Korabecna M, Calda P, Brestak M, Pazourkova E, Pospisilova S, Krkavcova M, Novotna M, Horinek A (2016). Differentially expressed miRNAs in trisomy 21 placentas. Prenat Diagn.

[30] Luo SS, Ishibashi O, Ishikawa G, Ishikawa T, Katayama A, Mishima T, Takizawa T, Shigihara T, Goto T, Izumi A, Ohkuchi A, Matsubara S, Takeshita T, Takizawa T (2009). Human villous trophoblasts express and secrete placenta-specific microRNAs into maternal circulation via exosomes. Biol Reprod.

[31] Chang G, Mouillet JF, Mishima T, Chu T, Sadovsky E, Coyne CB, Parks WT, Surti U, Sadovsky Y (2017). Expression and trafficking of placental microRNAs at the feto-maternal interface. FASEB J.

[32] Siew WH, Tan KL, Babaei MA, Cheah PS, Ling KH (2013). MicroRNAs and intellectual disability (ID) in down syndrome, X-linked ID, and Fragile X syndrome. Front Cell Neurosci.

[33] Xu Y, Li W, Liu X, Ma H, Tu Z, Dai Y (2013). Analysis of microRNA expression profile by small RNA sequencing in Down syndrome fetuses. Int J Mol Med.

[34] Lim JH, Lee DE, Kim SY, Kim HJ, Kim KS, Han YJ, Kim MH, Choi JS, Kim MY, Ryu HM, Park SY (2015). MicroRNAs as potential biomarkers for noninvasive detection of fetal trisomy 21. J Assist Reprod Genet.

[35] Zhang Y, Liao JM, Zeng SX, Lu H (2011). p53 downregulates down syndrome-associated DYRK1A through miR-1246. EMBO Rep.

[36] Faul F, Erdfelder E, Lang A-G, Buchner A (2007). G* Power 3: a flexible statistical power analysis program for the social, behavioral, and biomedical sciences. Behav Res Methods.

[37] Morales-Prieto DM, Ospina-Prieto S, Schmidt A, Chaiwangyen W, Markert UR (2014). Elsevier trophoblast research award lecture: origin, evolution and future of placenta miRNAs. Placenta.

[38] Morales-Prieto DM, Ospina-Prieto S, Chaiwangyen W, Schoenleben M, Markert UR (2013). Pregnancy-associated miRNA-clusters. J Reprod Immunol.

[39] Gu Y, Sun J, Groome LJ, Wang Y (2013). Differential miRNA expression profiles between the first and third trimester human placentas. AJP.

[40] Morales-Prieto DM, Chaiwangyen W, Ospina-Prieto S, Schneider U, Herrmann J, Gruhn B, Markert UR (2012). MicroRNA expression profiles of trophoblastic cells. Placenta.

[41] Higashijima A, Miura K, Mishima H, Kinoshita A, Jo O, Abe S, Hasegawa Y, Miura S, Yamasaki K, Yoshida A, Yoshiura K, Masuzaki H (2013). Characterization of placenta-specific microRNAs in fetal growth restriction pregnancy. Prenat Diagn.

[42] Salomon C, Guanzon D, Scholz-Romero K, Longo S, Correa P, Illanes SE, Rice GE (2017). Placental exosomes as early biomarker of preeclampsia: potential role of exosomal MicroRNAs across gestation. J Clin Endocrinol Metab.

[43] McDonald JS, Milosevic D, Reddi HV, Grebe SK, Algeciras-Schimnich A (2011). Analysis of circulating microRNA: preanalytical and analytical challenges. Clin Chem.

[44] Marabita F, de Candia P, Torri A, Tegner J, Abrignani S, Rossi RL (2016). Normalization of circulating microRNA expression data obtained by quantitative real-time RT-PCR. Brief Bioinform.

[45] Mestdagh P, Van Vlierberghe P, De Weer A, Muth D, Westermann F, Speleman F, Vandesompele J (2009). A novel and universal method for microRNA RT-qPCR data normalization. Genome Biol.

[46] Ferracin M, Lupini L, Salamon I, Saccenti E, Zanzi MV, Rocchi A, Da Ros L, Zagatti B, Musa G, Bassi C (2015). Absolute quantification of cell-free microRNAs in cancer patients. Oncotarget.

[47] Kotlabova K, Doucha J, Chudoba D, Calda P, Dlouha K, Hromadnikova I (2013). Extracellular chromosome 21-derived microRNAs in euploid & aneuploid pregnancies. Indian J Med Res.

[48] Zbucka-Kretowska M, Niemira M, Paczkowska-Abdulsalam M, Bielska A, Szalkowska A, Parfieniuk E, Ciborowski M, Wolczynski S, Kretowski A (2019). Prenatal circulating microRNA signatures of foetal down syndrome. Sci Rep.

[49] Noble WS (2009). How does multiple testing correction work?. Nat Biotechnol.

[50] Sourvinou IS, Markou A, Lianidou ES (2013). Quantification of circulating miRNAs in plasma: effect of preanalytical and analytical parameters on their isolation and stability. J Mol Diagn.

[51] Mestdagh P, Hartmann N, Baeriswyl L, Andreasen D, Bernard N, Chen C, Cheo D, D'Andrade P, DeMayo M, Dennis L, Derveaux S, Feng Y, Fulmer-Smentek S, Gerstmayer B, Gouffon J, Grimley C, Lader E, Lee KY, Luo S, Mouritzen P, Narayanan A, Patel S, Peiffer S, Ruberg S, Schroth G, Schuster D, Shaffer JM, Shelton EJ, Silveria S, Ulmanella U, Veeramachaneni V, Staedtler F, Peters T, Guettouche T, Wong L, Vandesompele J (2014). Evaluation of quantitative miRNA expression platforms in the microRNA quality control (miRQC) study. Nat Methods.

[52] Kowal J, Tkach M, Thery C (2014). Biogenesis and secretion of exosomes. Curr Opin Cell Biol.

[53] Ludwig AK, Giebel B (2012). Exosomes: small vesicles participating in intercellular communication. Int J Biochem Cell Biol.

[54] Salomon C, Ryan J, Sobrevia L, Kobayashi M, Ashman K, Mitchell M, Rice GE (2013). Exosomal signaling during hypoxia mediates microvascular endothelial cell migration and vasculogenesis. PLoS ONE.

[55] Sabapatha A, Gercel-Taylor C, Taylor DD (2006). Specific isolation of placenta-derived exosomes from the circulation of pregnant women and their immunoregulatory consequences. Am J Reprod Immunol.

[56] Redman CW, Sargent IL (2008). Circulating microparticles in normal pregnancy and pre-eclampsia. Placenta.

[57] Pidoux G, Gerbaud P, Cocquebert M, Segond N, Badet J, Fournier T, Guibourdenche J, Evain-Brion D (2012). Review: Human trophoblast fusion and differentiation: lessons from trisomy 21 placenta. Placenta.

[58] Salvi A, Vezzoli M, Busatto S, Paolini L, Faranda T, Abeni E, Caracausi M, Antonaros F, Piovesan A, Locatelli C, Cocchi G, Alvisi G, De Petro G, Ricotta D, Bergese P, Radeghieri A (2019). Analysis of a nanoparticleenriched fraction of plasma reveals miRNA candidates for down syndrome pathogenesis. Int J Mol Med.

[59] Maccani MA, Avissar-Whiting M, Banister CE, McGonnigal B, Padbury JF, Marsit CJ (2010). Maternal cigarette smoking during pregnancy is associated with downregulation of miR-16, miR-21, and miR-146a in the placenta. Epigenetics.

[60] Hromadnikova I, Kotlabova K, Doucha J, Dlouha K, Krofta L (2012). Absolute and relative quantification of placenta-specific micrornas in maternal circulation with placental insufficiency-related complications. J Mol Diagn.

