# Supplementary material for: Genome-wide miRNA profiling in plasma of pregnant women with down syndrome fetuses
Source: Mol Biol Rep. 2020 May 30;47(6):4531–40. doi: 10.1007/s11033-020-05545-w (PMC7295716; doi:10.1007/s11033-020-05545-w)
Supplement: Supplementary file 1 — Supplementary file1 (DOC 33 kb) [file 11033_2020_5545_MOESM1_ESM.doc]

**Optimisation of the miRNA extraction**

We tested six different miRNA isolation kits: NucleoSpin miRNA Plasma (Macherey-Nagel, Germany), miRCURY RNA Isolation Kit – Biofluids (Exiqon, Denmark), miRNeasy Serum/Plasma (Qiagen, Germany), mirVana PARIS (Ambion, Life Technologies, USA), QIAamp Circulating Nucleic Acid (Qiagen) and PME microRNAs Extraction Kit (Analytic Jena, Germany). Various input (200–2000 μl) and elution (14–50 μl) volumes were tested for each kit. Improvement of miRNA extraction using a vacuum concentrator (SpeedVac, Thermo Fisher Scientific) or glycogen (Roche Diagnostics, USA and Invitrogen, Thermo Fisher Scientific, USA) were also tested. Concentration and quality of isolated miRNA samples were then evaluated using three different approaches:

1) fluorometer (Qubit 3.0) with microRNA Assay Kit (Thermo Fisher Scientific),

2) spectrophotometer (IMPLEN, Germany), and

3) quantitative reverse transcription PCR (RT-qPCR) with TaqMan MicroRNA Reverse Transcription Kit and selected TaqMan microRNA assays using QuantStudio 12K Flex (Applied Biosystems).

The four best variants of sample processing were compared directly on the array strip before the pilot study. The best combination was selected for the clinical sample preparation.

Testing of Isolation kits

While the miRNeasy Serum/Plasma Kit with 200 μl input of plasma was found to be the most efficient, the highest absolute yields were achieved with the NucleoSpin miRNA Plasma Kit and miRCURY RNA Isolation Kit with an input of 900 μl of plasma in both cases. These three isolation procedures were further improved using a vacuum concentrator (SpeedVac) and glycogen addition (2 μg and 10 μg; Roche and Invitrogen) when merging two isolations (miRNeasy 400 μl; NucleoSpin and miRCURY 1800 μl overall input volume) with default elution volumes (miRNeasy 2 x 14 μl; NucleoSpin and miRCURY 2 x 20 μl).

Glycogen and vacuum concentrator

Glycogen from both tested suppliersreached comparable results. Higher isolation yields were achieved with 2 μg of glycogen then with 10 μg. While the addition of 2 μg of glycogen increased the total miRNA concentrations, as measured with a fluorometer, the increase differed between individual miRNA concentrations as measured with RT-qPCR. Therefore, the use of glycogen was excluded, because it seemed to affect the overall miRNA profile.

When using the SpeedVac, miRNA concentration increases as measured with a fluorometer always exactly corresponded to the increases in plasma input volumes. However, inconsistent results were reported in the quantification of individual miRNA assays by qPCR. In some assays, samples concentrated with SpeedVac improved as expected – Ct (cycle threshold) values decreased approximately by one cycle. Nevertheless, in other assays, the Ct values of concentrated samples increased in some cases by two or three cycles. This indicates elevated levels of PCR inhibitors in samples where the vacuum concentrator was used. Increases in inhibitor concentrations can decrease the efficiency of PCR amplification with a varying impact depending on absolute miRNA concentration, with a higher impact on the less-represented miRNAs.

Finally, we compared miRNAs isolated using NucleoSpin and miRCURY, with (1800 μl input; 2 x 20 μl elution volumes) and without (900 μl input; 20 μl elution volumes) using the SpeedVac directly on the miRNA array strip. The output readings of both samples adjusted with SpeedVac flagged an inhibition of the hybridisation procedure, so the use of the SpeedVac was also excluded. Since the sample isolated with NucleoSpin kit achieved slightly better results (higher overall fluorescence signal) than the sample isolated with miRCURY, it was selected for isolation of clinical samples included in our study.
