# Supplementary material for: Genome-wide miRNA profiling in plasma of pregnant women with down syndrome fetuses
Source: Mol Biol Rep. 2020 May 30;47(6):4531–40. doi: 10.1007/s11033-020-05545-w (PMC7295716; doi:10.1007/s11033-020-05545-w)
Supplement: Supplementary file 2 — Supplementary file2 (DOC 16 kb) [file 11033_2020_5545_MOESM2_ESM.doc]

| **miRNA** | **Power** |
| --- | --- |
| let-7a | 1.000 |
| miR-107 | 1.000 |
| miR-10b | 1.000 |
| miR-122 | 1.000 |
| miR-1246 | 1.000 |
| miR-125b | 1.000 |
| miR-193a | 1.000 |
| miR-3613 | 1.000 |
| miR-378a | 1.000 |
| miR-498 | 1.000 |
| miR-500a | 1.000 |
| miR-615 | 1.000 |
| miR-654 | 1.000 |
| miR-6891 | 1.000 |
| miR-940 | 1.000 |
| let-7d | 1.000 |
| miR-4668 | 1.000 |
| miR-6511 | 1.000 |
| let-7c | 0.996 |
| miR-103a | 0.995 |
| miR-99a | 0.984 |
| miR-542 | 0.103 |
